# Research on Underwater Constant High-Voltage DC Switching Technology for MCSEM

**DOI:** 10.3390/s24206598

**Published:** 2024-10-13

**Authors:** Zhibin Ren, Meng Wang, Xianhu Luo, Chentao Wang, Tailong Chen

**Affiliations:** 1 School of Geophysics and Information Technology, China University of Geosciences Beijing, Beijing 100083, China; rzb@email.cugb.edu.cn (Z.R.); 3010200009@cugb.edu.cn (C.W.); ctl@email.cugb.edu.cn (T.C.); 2 State Key Laboratory of Geological Processes and Mineral Resources, China University of Geosciences Beijing, Beijing 100083, China; 3 Guangzhou Marine Geological Survey, Guangzhou 511458, China

**Keywords:** marine controlled source electromagnetic, constant high-voltage, DC switching inverter, SPWM control

## Abstract

The marine controlled source electromagnetic (MCSEM) transmitter can transmit high currents near the seabed to detect the electrical structure of the seafloor. The use of three-phase alternating current (AC) transmission can lead to three-phase imbalance, which results in an excessive current in one phase’s power line and affects the safety of the tow cable. This paper proposes an MCSEM underwater constant high-voltage direct-current (DC) switching scheme that replaces AC transmission with DC transmission. This scheme can fundamentally avoid three-phase imbalance and the AC loss caused by inductance. After establishing a simulation model to analyze the effect of the scheme, the relevant hardware units were designed. The hardware unit mainly consists of three parts: a DC switching inverter unit, a filter unit, and a step-down rectification unit. The DC inverter unit controls six insulated gate bipolar transistor (IGBT) modules with sinusoidal pulse width modulation (SPWM) signals to convert DC to three-phase AC power; the filter unit filters out extra harmonic components; and the step-down rectification unit converts high-voltage three-phase AC to low-voltage DC. The scheme ultimately achieved an adjustable DC output of 48.3–73.4 V under a constant DC input voltage of 3000 V and effectively reduced the current on the cable. This scheme has the potential to replace the previous AC transmission, reducing the risk of tow cable burnout and enhancing the safety of MCSEM operations.

## 1. Introduction

**Figure 1:**
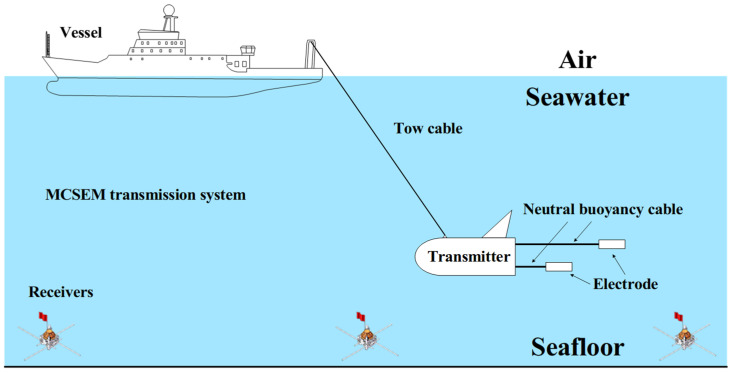
A schematic diagram of marine controlled source electromagnetic detection.

Marine controlled source electromagnetic (MCSEM) detection is an effective technique for the detection of resistivity anomalies such as seafloor natural gas hydrate resources [1,2,3,4,5]. As shown in Figure 1, the vessel tows an electromagnetic transmitter near the seafloor via a long tow cable, which transmits high-power electromagnetic waves to the seafloor [6,7,8]. The reflected electromagnetic data can be recorded by receivers deployed on the seafloor in advance, and the electrical distribution characteristics of the sub-seafloor medium can be deduced after data processing [9,10,11,12]. This method can effectively compensate for the lack of high frequencies in natural electromagnetic field sources.

According to David’s calculations of the electromagnetic field distribution [13], it is evident that, with other parameters held constant, increasing the transmitter output current can result in a larger response generated by the horizontal electric dipole source on the seafloor. Increasing the transmitter output current implies an increase in the current on the tow cable. The tow cable is the only channel connecting the deck end and the underwater end, and the maximum current that it can transmit determines the maximum transmitter output current of the transmission system. This is currently one of the factors limiting the excitation capabilities of artificial electromagnetic field sources. The current MCSEM transmission systems commonly use alternating-current (AC) transmission (single-phase or three-phase), and there is AC loss on the tow cable caused by resistance and inductance. As the current transmitted through the tow cable increases, the AC loss also increases [14]. To reduce the power loss on the tow cable, the transmission voltage is usually increased when the transmitted power is constant. As an example, the Deep Blue transmission system from EMGS in Norway has a transmission voltage of 24,000 V AC at a high voltage, with transmission power of 1350 kW, and realizes excitation with a high current of 10,000 A [15]. However, its scheme places an extremely high demand on both the vessel and the tow cable, which is difficult to meet. In addition, there is an unavoidable three-phase imbalance in MCSEM transmission systems that use three-phase AC transmission. Taking the China University of Geosciences (Beijing) as an example, in 2019, they developed a transmission system based on a 32.8 mm photoelectric composite cable, achieving a maximum transmitter output current of 1988 A [16]. The transmitter used a transformer with a 30:1 ratio. Under normal conditions, when the transmitter load current reaches 1500 A, the current transmitted through the tow cable should be approximately 37 A. However, due to the presence of the three-phase imbalance, the maximum current in one phase of the tow cable approached 40 A. When the transmitter load current exceeded 1900 A, the current in the tow cable increased further. Therefore, the transmission system could only operate at 1988 A for a short period and could not sustain prolonged operation, otherwise facing the risk of burning the tow cable.

The tow cable exhibits both resistance and inductance, and the loss caused by resistance is inevitable. However, the inductance has a hindering effect on the alternating current, while it has a negligible effect on the direct current (DC). Inspired by long-distance high-voltage direct-current transmission technology for both land and sea [17,18], the MCSEM transmission system can be optimized by modifying it to use DC transmission. By maintaining the transmission voltage on the tow cable at its maximum allowable value, the tow cable can operate in a minimum current mode. This can fundamentally avoid the issue of three-phase imbalance [19] and high AC losses, thereby enhancing the transmission security and maximizing the transmission potential of the tow cable. In the process of achieving DC power transmission from the deck end to the underwater end, the design of an underwater high-voltage DC switching system is an indispensable and critical step.

## 2. Analysis of Three-Phase AC MCSEM Transmission System

**Figure 2:**
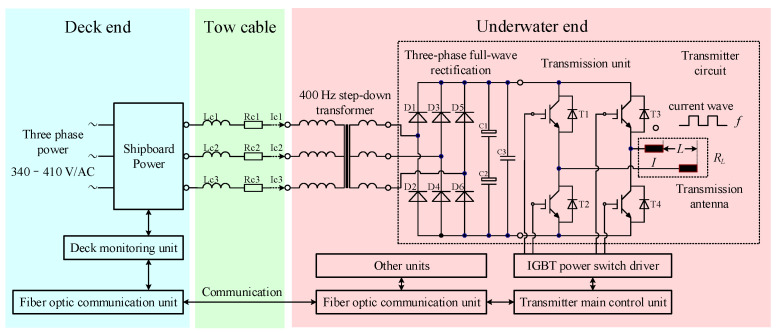
A schematic diagram of the three-phase AC transmission system. Lc1, Lc2, and Lc3 are the inductances on the three-phase power lines of the tow cable. Rc1, Rc2, and Rc3 are the resistances on the three-phase power lines of the tow cable. Ic1, Ic2, and Ic3 are the currents transmitted on the three-phase power lines of the tow cable. D1, D2, D3, D4, D5, and D6 are rectifier diodes. C1, C2, and C3 are filter capacitors. T1, T2, T3, and T4 are insulated gate bipolar transistor modules.

The schematic diagram of the MCSEM transmission system using three-phase AC transmission is shown in Figure 2. The ship-borne high-power supply outputs 400 Hz three-phase electricity, which is transmitted to the underwater transmitter via a tow cable. The three-phase electricity is stepped down by a transformer and then rectified by a three-phase full-wave rectifier to obtain DC power for the transmission unit.

The excitation capability of the artificial field source depends on several parameters [20,21,22], including the transmission frequency *f*, transmitter output current *I*, and transmission electrode distance *L*. Under the condition of a certain acquisition level, a greater excitation capability of the artificial source means a better signal-to-noise ratio and deeper detection depth. Under the condition of a certain transmission frequency *f*, increasing the excitation capability of the artificial field source means increasing the product of the transmitter output current *I* and the transmission electrode distance *L*. The best way to increase the product *I* × *L* is to simultaneously increase both *I* and *L*. When *L* increases, the equivalent resistance *R_L_* between the transmission antennae will also increase. Therefore, the transmitter’s output power *P* = *I*^2^*R_L_* will increase, and the tow cable will transmit greater power from the deck end. As the current transmitted on the tow cable increases, the AC loss caused by inductance will also increase. Additionally, three-phase AC transmission faces the issue of three-phase imbalance. During transformer manufacturing, it is impossible to achieve perfectly symmetrical impedance characteristics in the transformer’s windings. Similarly, the inductance Lc1, Lc2, Lc3 and resistance Rc1, Rc2, Rc3 of the power lines in the tow cable cannot be made perfectly consistent. This leads to a current imbalance in the three-phase power lines during actual operation. An excessive current in any phase can affect the safety of the tow cable and transformer. The three-phase imbalance is one of the main limiting factors for MCSEM’s safe operation.

In traditional transmission system design, the load of the transmitter is first calculated. Based on this load and the expected transmitter load current, an appropriate transformer ratio is selected to determine the maximum output voltage of the high-power supply. The transmitter’s load is closely related to the transmission electrode. Given that achieving ideal conditions in the processing and use of transmission electrodes is difficult, the actual load often exceeds the calculated value. This typically results in the final transmitter load current being lower than expected, leading to suboptimal detection performance. This is another problem faced by AC MCSEM transmission systems.

## 3. MCSEM Underwater High-Voltage DC Power Switching Scheme

**Figure 3:**
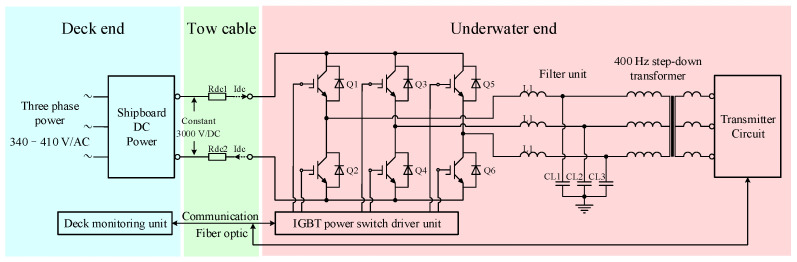
A schematic diagram of MCSEM underwater high-voltage DC transmission.

Due to the issues of high AC loss and three-phase imbalance in AC transmission, this paper proposes an MCSEM underwater high-voltage DC power switching scheme. Figure 3 shows a schematic diagram of MCSEM underwater high-voltage DC transmission. Compared to the AC transmission scheme, the output of the ship-borne high-power supply has been changed to a high-voltage DC 3000 V output. The loss on the tow cable is only the DC loss caused by the resistance, and the three-phase imbalance is avoided. The high-voltage DC transmitted underwater first passes through a DC switching inverter unit composed of six insulated gate bipolar transistor (IGBT) modules (Q1–Q6) and is converted into three sets of sinusoidal pulse width modulation (SPWM) waves [23]. The SPWM wave then passes through the filter unit [24] and is converted into three-phase AC. The three-phase AC is stepped down by a 400 Hz transformer and rectified by a three-phase full-wave rectifier to obtain a DC voltage for the transmitter. Adjusting the SPWM wave parameters can produce the variable DC voltage.

### 3.1. DC Switching Unit

**Figure 4:**
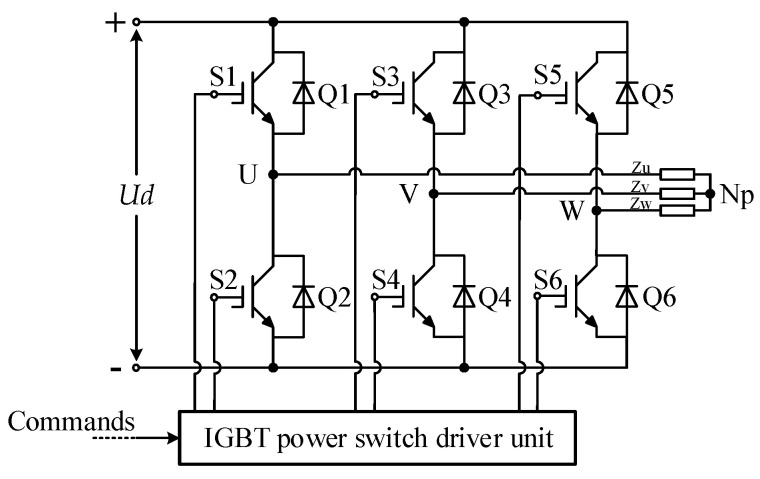
A schematic diagram of the DC switching inverter unit.

In the MCSEM DC transmission scheme, the DC high-voltage output from the ship-borne high-power supply needs to be stepped down before it can be supplied to the transmitter. Since the transformer can only step down AC, the DC power transmitted to the underwater end needs to be converted to AC power. This paper uses a three-phase full-bridge inverter circuit as the DC switching inverter unit, as shown in Figure 4 [25,26].

The DC switching inverter unit is composed of 6 IGBT modules (Q1–Q6) in total. Q1, Q2, Q3, and Q4, as well as Q5 and Q6, form the upper and lower bridge arms of the three IGBT bridges, respectively. The IGBT power switch driver unit outputs 6 control signals (S1–S6) to control the switching on and off of the IGBT modules. At any moment, each set of IGBT bridges allows only one of the upper or lower bridge arms to be open. Therefore, S1 is complementary to S2, S3 is complementary to S4, and S5 is complementary to S6. The outputs of points U, V, and W are all SPWM waves. Zu, Zv, and Zw represent the impedance on the three-phase power lines, and Np is the hypothetical neutral point.

### 3.2. SPWM Control Method

**Figure 5:**
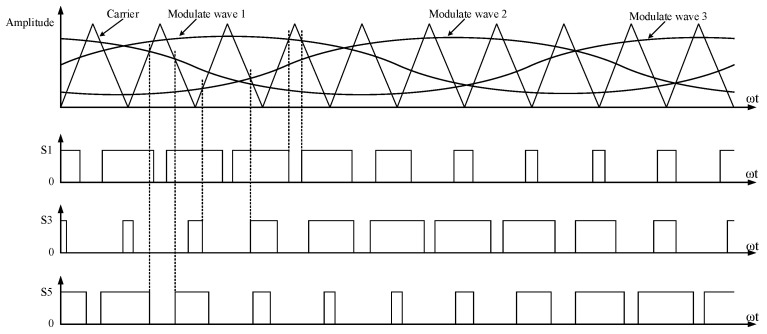
Diagram of the generation of SPWM control signals.

SPWM technology can generate pulse widths that vary according to a sinusoidal pattern [27]. The area of the output pulse voltage within a given interval is equal to the area of the desired sine wave. This technology uses a triangular wave as the carrier wave and a sine wave as the modulation wave. Within one cycle, when the amplitude of the sine wave is greater than that of the triangular wave, the output is high; otherwise, the output is low [28]. Figure 5 shows the generation process of SPWM control signals. Three sine waves, each with a 120° phase difference from each other, are compared with a triangular wave to obtain three SPWM control signals.

The pulse width time obtained using the symmetric rule-based sampling method [29,30] is given by the following formula:

(1) $$Ton=\frac{1}{2Nf_{m}}\left(1+m\sin\omega_{m}t\right)$$

(2) $$N=\frac{f_{c}}{f_{m}}$$

(3) $$m=\frac{A_{m}}{A_{c}}$$

where *N* is the carrier ratio, which is the ratio of the frequency of the carrier wave (triangular wave) to the frequency of the modulation wave (sine wave); *m* is the modulation index, which is the ratio of the amplitude of the modulation wave to the amplitude of the carrier wave; *f_m_* is the frequency of the modulation wave; *f_c_* is the frequency of the carrier wave; *A_m_* is the amplitude of the modulation wave; *A_c_* is the amplitude of the carrier wave; and *ω_m_* is the angular frequency of the modulation wave, where *ω_m_* = 2π*f_m_*. As *N* increases, the pulse width decreases; as *m* increases, the pulse width increases. In an ideal situation, as the value of *N* increases, the number of modulation pulses increases, and the width variation of the SPWM pulses becomes closer to the sine wave. However, an increasing *N* will lead to a higher switching frequency for the IGBT, resulting in a greater switching loss. Therefore, *N* is generally chosen to be between 5 and 10.

The phase voltage *Uu* and the fundamental components of the line voltage *Uuv*_1_ are given by the following formulas:

(4) $$Uu=\frac{Ud}{2}\left\{m\sin\omega_{m}t+\sum_{n=1}^{\infty }\frac{4}{n\pi }\sin\left[\frac{mn\pi }{2}\sin\left(\omega_{m}t\right)+\frac{n\pi }{2}\right]\cos\left(n\omega_{c}t\right)\right\}$$

(5) $$Uuv_{1}=\frac{\sqrt{3}Ud}{2}m\sin\left(\omega_{m}t-\frac{\pi }{3}\right)$$

where *Ud* is the DC input voltage, *n* is the harmonic number, and *ω_c_* is the angular frequency of the modulation wave. As *m* increases, the output voltage of the three-phase inverter unit also increases. However, when *m* > 1, overmodulation will occur, leading to waveform distortion and higher harmonic distortion. Therefore, *m* generally does not exceed 1.

## 4. Simulation Analysis of DC Switching Inverter

**Figure 6:**
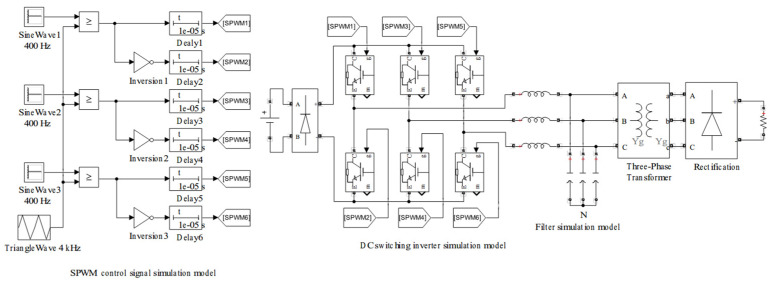
The underwater high-voltage DC switching simulation model.

Based on the previously proposed underwater high-voltage DC power switching scheme, a corresponding model is established using the Simulink tool in the MATLAB R2013b software. Figure 6 shows the underwater high-voltage DC switching simulation model.

**Figure 7:**
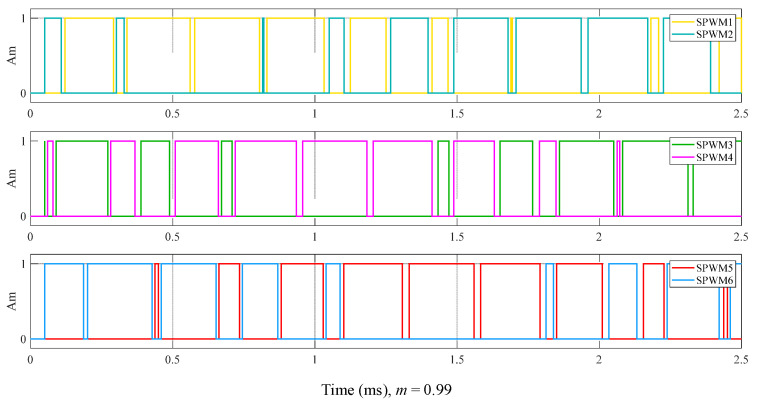
The simulation result for six SPWM control signals when *m* = 0.99.

**Figure 8:**
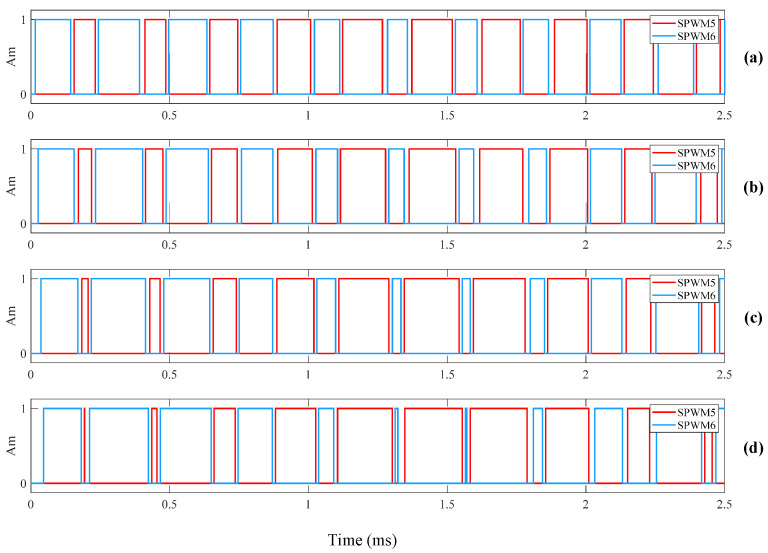
The simulation result of the SPWM signals for different values of *m*. (**a**) *m* = 0.3; (**b**) *m* = 0.5; (**c**) *m* = 0.7; (**d**) *m* = 0.9.

The SPWM control signal simulation model is configured with three sine wave generators and one triangular wave generator. All three sine waves have a frequency of 400 Hz, matching the operating frequency of the underwater transformer, with a phase difference of 120° between each other. To obtain a control signal that more closely follows the sine wave pattern, *N* is set to 10. The triangular wave frequency is 4 kHz. Comparing the sine wave with the triangular wave generates the upper bridge arm control signals SPWM1, SPWM3, and SPWM5. Inverting the upper bridge arm control signals generates the lower bridge arm control signals SPWM2, SPWM4, and SPWM6. To prevent the simultaneous switching on of the upper and lower arms of the DC switching inverter unit [31], the rising edges of all control signals are delayed by 10 µs to obtain the final control signals. This delay is the dead time of the control signals [32]. Figure 7 shows the simulation result of the six control signals when *m* = 0.99. To investigate the effect of *m* on the control signals, different values of *m* were set to observe the control signals. The values of *m* were set to 0.3, 0.5, 0.7, and 0.9 to observe the variations in the SPWM5 and SPWM6 signals. Figure 8 shows the simulation result of the SPWM signals for different values of *m*. It can be observed that as *m* increases, the variations in the density of the SPWM control signals become more pronounced.

**Figure 9:**
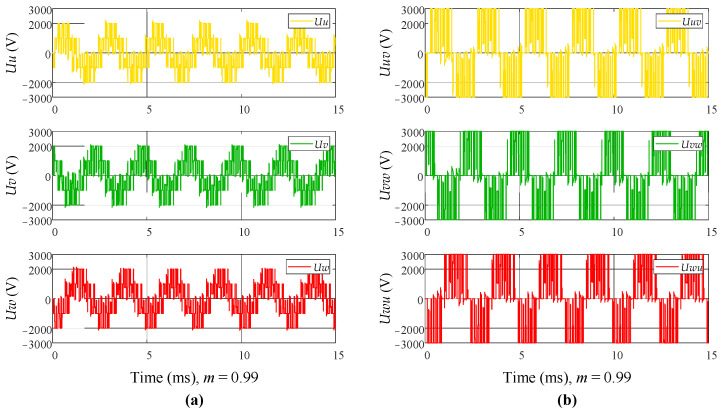
The simulation result of the DC switching inverter output when *m* = 0.99. (**a**) Phase voltage; (**b**) line voltage.

**Figure 10:**
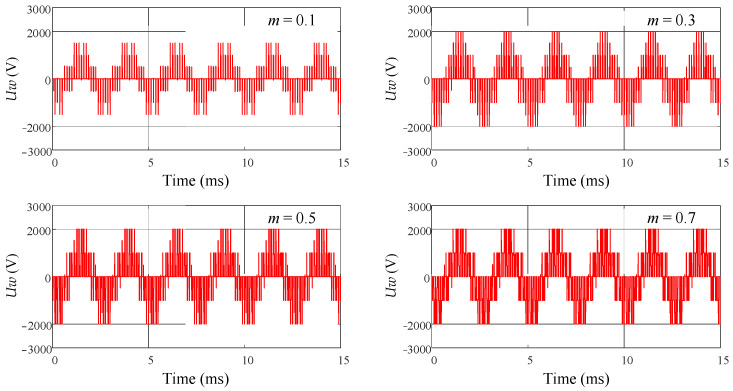
The simulation result of phase voltage *Uw* for different values of *m*.

**Figure 11:**
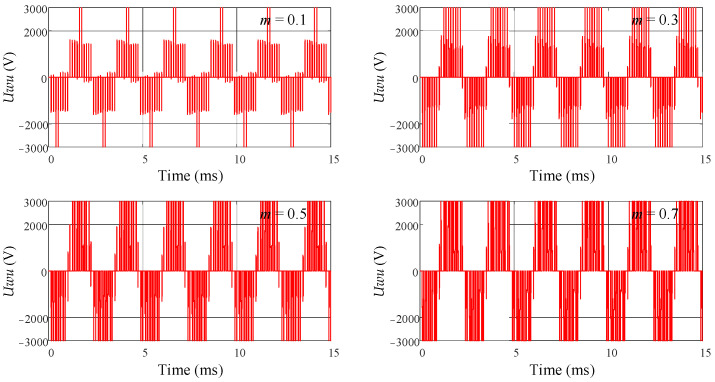
The simulation result of line voltage *Uwu* for different values of *m*.

**Figure 12:**
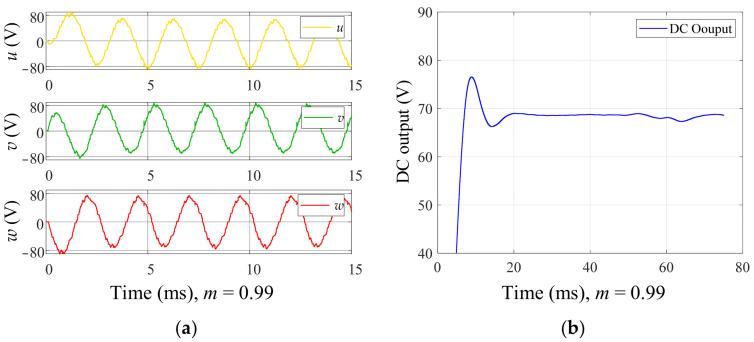
(**a**) The simulation result of the line voltage at the transformer output; (**b**) the simulation result of the DC output voltage after rectification.

The six control signals are connected to the DC switching inverter unit, and the output of the DC switching inverter is observed. The DC supply voltage is set to 3000 V. Figure 9 shows the simulation result of the DC switching inverter unit when *m* = 0.99. It can be observed that the phase voltage exhibits the effect of voltage superposition and follows a sinusoidal pattern. Additionally, *m* is set to different values to observe its impact on the phase voltage and line voltage. Figure 10 shows the simulation result of the phase voltage *Uw* for different values of *m*, and Figure 11 shows the simulation result of the line voltage *Uwu* for different values of *m*. It can be observed that as the value of *m* increases, the short pulses in the phase and line voltages become denser. Figure 12a shows the simulation result of the line voltage at the transformer output, and the line voltages are sinusoidal waves. Figure 12b shows the simulation result of the DC output voltage at the transformer output after rectification. It can be observed that, after three-phase full-wave rectification, a slightly fluctuating DC voltage is obtained.

## 5. Test

### 5.1. SPWM Control and DC Output Test

**Figure 13:**
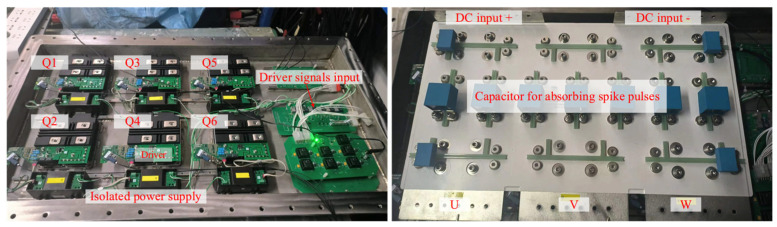
The DC switching inverter unit.

Based on the MCSEM underwater high-voltage DC power switching scheme proposed in this paper, a DC switching inverter unit was designed, as shown in Figure 13. Table 1 shows the main components and key parameters in the test.

**Figure 14:**
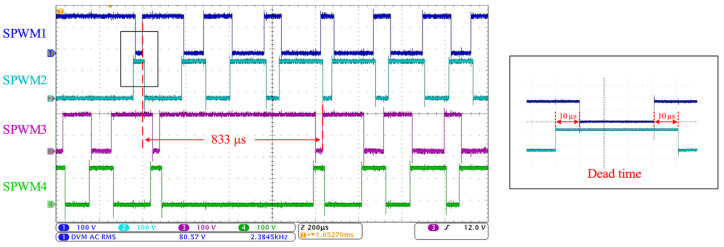
The test result of the SPWM control waveform and dead time (Q1–Q4).

A laminated busbar connects the IGBT modules, and several capacitors are placed to absorb the spike pulses generated by the IGBT switching. The control signals SPWM1–SPWM4 of Q1–Q4 are shown in Figure 14. SPWM1 is inverted relative to SPWM2, and SPWM3 is inverted relative to SPWM4. SPWM3 is delayed by 833 μs relative to SPWM1, which is one-third of 2.5 ms, corresponding to a phase difference of 120°. Each edge of the signal has a dead time of 10 μs. The test result of the control signals for Q3–Q6 is consistent with the measurement result of the control signals for Q1–Q4. The phase difference between SPWM1, SPWM3, and SPWM5 is 120° relative to each other.

**Figure 15:**
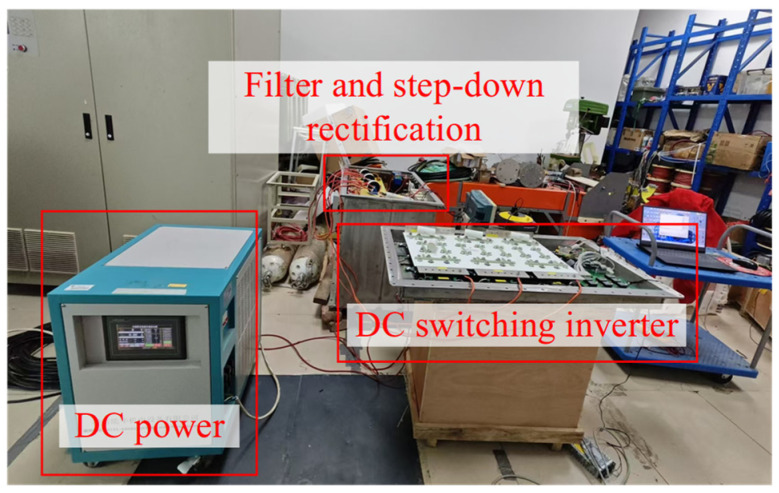
Actual test scenario diagram of DC power switching scheme.

**Figure 16:**
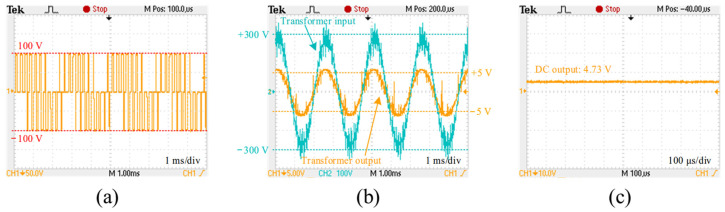
Test result. (**a**) The output voltage between any two phases of the DC switching inverter unit; (**b**) the line voltage at the transformer input and output; (**c**) the DC output.

The DC switching inverter unit was then connected with the filter unit and the three-phase full-wave rectifier unit for the overall test of the DC power switching scheme, as shown in Figure 15. The six-channel SPWM control signals were input into the DC switching inverter unit, and the voltage output between any two phases was observed using an oscilloscope. For safety, the DC input voltage was set to 100 V, with *m* set to 0.5. The actual line voltage waveform between any two phases, as shown in Figure 16a, was essentially consistent with the simulation result, indicating that the DC switching inverter unit was operating normally. After this, the DC voltage input was set to 300 V and *m* was set to 0.5, to test the transformer and the three-phase rectification unit. Figure 16b shows the test result of the line voltage between any two phases at the transformer input and output end. It can be observed that the transformer input and output are sine waves. Figure 16c shows the test result of the DC output after three-phase rectification, with a DC output of 4.73 V. This demonstrates that the DC power switching scheme proposed in this paper can achieve a stable DC output.

**Figure 17:**
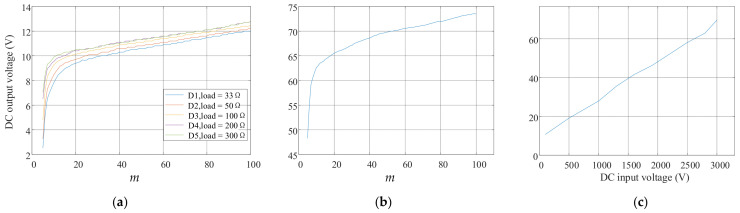
(**a**) The DC output voltage test result under different loads (DC input = 500 V); (**b**) the test result of DC output voltage variation with *m* under 3000 V DC input condition; (**c**) the test result of DC output voltage variation with DC input.

Subsequently, the DC input voltage was set to 500 V to test the impact of the modulation index *m* on the final DC output voltage, as shown in Figure 17a. The modulation index *m* ranged from 0.01 to 0.99. D1 to D5 represent the test results of the DC output voltages under load conditions of 33 Ω, 50 Ω, 100 Ω, 200 Ω, and 300 Ω, respectively. It can be observed that, with other conditions remaining constant, as *m* increases, the DC output voltage also increases. The DC output voltage varies non-linearly with changes in *m*, and the rate of change will experience a shift near *m* = 0.1. When *m* > 0.2, the DC output voltage increases approximately linearly with *m*. Then, the DC input voltage was increased to 3000 V to verify the effectiveness of the MSCEM DC power switching scheme under a high input voltage. The test result is shown in Figure 17b. When *m* is 0.05, the DC output is 48.3 V; when *m* is 0.99, the DC output is 73.4 V. This variation process proved that the DC power switching scheme successfully achieved the adjustability of the DC output voltage. Finally, with *m* fixed at 0.99, the DC input voltage was gradually increased from a low voltage to 3000 V, to observe the changes in the DC output. Figure 17c shows the test result of the DC output voltage variation with the DC input voltage. The DC output voltage exhibits good linearity with changes in the DC input voltage. The performance of the entire MCSEM high-voltage DC power switching scheme is relatively stable.

### 5.2. DC Power Supply Output Current Test

On the basis of achieving an adjustable voltage, the hardware unit of the DC power supply switch scheme was connected to the transmission unit to verify the effectiveness of the scheme. The test was conducted by adjusting the supply voltage on the DC side and the SPWM control signals to keep the transmitter load current constant and observing the changes in the output current on the DC side. Table 2 shows the test data of DC transmission under different load currents. *I* is the transmitter load current, *Ud* is the DC power supply voltage, *Id* is the DC power supply output current, and *m* is the modulation index of the SPWM control signals. It can be observed that increasing the DC-side voltage while keeping the transmitter load current constant can reduce the current transmitted through the cable. However, this also leads to higher total DC power consumption. The increase in the total power consumption is primarily due to two factors: first, the efficiency of SPWM control decreases as *m* increases; second, the harmonic components input to the transformer cause additional losses. Table 3 shows the test data of DC transmission under different load currents. It can be seen that, even without a long-distance tow cable, the three-phase imbalance still exists. Comparing the test results of DC and AC transmission, increasing the transmission voltage has the capability to significantly reduce the current on the cable in the DC transmission scheme below that on the cable in the AC transmission scheme. Additionally, the DC power switching scheme can be further optimized in terms of the control strategies and circuit topology to achieve better results in the future.

## 6. Conclusions

This paper proposes an MCSEM underwater high-voltage DC power switching scheme and describes the related hardware units. The simulation analysis has verified the feasibility of the designed circuit topology, while actual testing has confirmed the scheme’s effectiveness.

This scheme uses DC transmission, which eliminates the AC loss and three-phase imbalance phenomenon. Under the condition of a constant DC input voltage of 3000 V, this scheme achieves an adjustable output DC output voltage within the range of 48.3 V to 73.4 V by changing the modulation index *m* (0.05–0.99). This allows for the convenient adjustment of the output voltage to maintain a stable transmitter output current according to real-time changes in the load. The test results show that the DC scheme can significantly reduce the transmission current on the cable by increasing the transmission voltage.

This scheme has the potential to replace the previous AC transmission, reducing the risk of tow cable burnout and enhancing the safety of MCSEM operations.

## Figures and Tables

**Table 1:** The characteristic parameters of the main components in the test.

Component	Characteristic Parameters
IGBT	V_CES_ = 1200 V, I_C_ = 2400 A
Laminated busbar	V_rated_ = 4000 V, I_rated_ = 30 A
Transformer	50:1
Inductor	22.5 mH
Capacitor	7 μF

**Table 2:** The test data of DC transmission under different load currents.

*I*	*Ud*	*Id*	*m*
36.3 A	220 V	0.87 A	0.99
36.2 A	300 V	0.70 A	0.5
36.3 A	410 V	0.56 A	0.3
36.3 A	1290 V	0.29 A	0.1
53.2 A	250 V	1.17 A	0.99
53.1 A	355 V	0.86 A	0.5
53.1 A	500 V	0.68 A	0.3
53.4 A	1550 V	0.36 A	0.1
67.8 A	270 V	1.41 A	0.99
67.7 A	398 V	1.01 A	0.5
67.6 A	570 V	0.78 A	0.3
67.7 A	1758 V	0.40 A	0.1
96 A	330 V	1.9 A	0.99
95.8 A	473.5 V	1.29 A	0.5
96 A	683 V	0.95 A	0.3
95.8 A	2110 V	0.46 A	0.1

**Table 3:** The test data of AC transmission under different load currents.

AC Voltage/V	Three-Phase Current/V			Load Current/A
	A	B	C	
171.1	0.51	0.79	0.47	36.3
198.2	0.70	1.17	0.67	53.0
221.2	0.92	1.53	0.83	67.9
260.2	1.29	2.17	1.07	95.9

## Data Availability

Data are contained within the article.

## List of Abbreviations

MCSEM	marine controlled source electromagnetic
AC	alternating current
DC	direct current
IGBT	insulated gate bipolar transistor
SPWM	sinusoidal pulse width modulation
